# Global Assessment of the Impact of Type 2 Diabetes on Sleep through Specific Questionnaires. A Case-Control Study

**DOI:** 10.1371/journal.pone.0157579

**Published:** 2016-06-17

**Authors:** Albert Lecube, Enric Sánchez, Fernando Gómez-Peralta, Cristina Abreu, Joan Valls, Olga Mestre, Odile Romero, María Dolores Martínez, Gabriel Sampol, Andreea Ciudin, Cristina Hernández, Rafael Simó

**Affiliations:** 1 Endocrinology and Nutrition Department, Hospital Universitari Arnau de Vilanova, Biomedical Research Institute of Lleida (IRBLLEIDA), Universitat de Lleida, Av Rovira Roure 80, 25198, Lleida, Spain; 2 CIBER de Diabetes y Enfermedades Metabólicas Asociadas (CIBERDEM), Instituto de Salud Carlos III (ISCIII), Diabetes and Metabolism Research Unit, Institut de Recerca i Hospital Universitari Vall d’Hebron (VHIR), Universitat Autònoma de Barcelona, Pg Vall d’Hebron 119-129, 08035, Barcelona, Spain; 3 Endocrinology and Nutrition Unit, Segovia General Hospital, C/Miguel Servet s/n, 40002, Segovia, Spain; 4 Biostatistics and Epidemiology Unit, Biomedical Research Institute of Lleida (IRBLLEIDA), Av Rovira Roure 80, 25198, Lleida, Spain; 5 CIBER Enfermedades Respiratorias (CIBERES), Instituto de Salud Carlos III (ISCIII), Sleep Unit, Neurophysiology Department, Institut de Recerca i Hospital Universitari Vall d’Hebron (VHIR), Universitat Autònoma de Barcelona, Pg Vall d’Hebron 119-129, 08035, Barcelona, Spain; 6 CIBER Enfermedades Respiratorias (CIBERES), Instituto de Salud Carlos III (ISCIII), Sleep Unit, Pneumology Department, Institut de Recerca i Hospital Universitari Vall d’Hebron (VHIR), Universitat Autònoma de Barcelona, Pg Vall d’Hebron 119-129, 08035, Barcelona, Spain; Hospital Universitario de La Princesa, SPAIN

## Abstract

**Abstract:**

Type 2 diabetes (T2D) is an independent risk factor for sleep breathing disorders. However, it is unknown whether T2D affects daily somnolence and quality of sleep independently of the impairment of polysomnographic parameters.

**Material and Methods:**

A case-control study including 413 patients with T2D and 413 non-diabetic subjects, matched by age, gender, BMI, and waist and neck circumferences. A polysomnography was performed and daytime sleepiness was evaluated using the Epworth Sleepiness Scale (ESS). In addition, 135 subjects with T2D and 45 controls matched by the same previous parameters were also evaluated through the Pittsburgh Sleep Quality Index (PSQI) to calculate sleep quality.

**Results:**

Daytime sleepiness was higher in T2D than in control subjects (p = 0.003), with 23.9% of subjects presenting an excessive daytime sleepiness (ESS>10). Patients with fasting plasma glucose (FPG ≥13.1 mmol/l) were identified as the group with a higher risk associated with an ESS>10 (OR 3.9, 95% CI 1.8–7.9, p = 0.0003). A stepwise regression analyses showed that the presence of T2D, baseline glucose levels and gender but not polysomnographic parameters (i.e apnea-hyoapnea index or sleeping time spent with oxigen saturation lower than 90%) independently predicted the ESS score. In addition, subjects with T2D showed higher sleep disturbances [PSQI: 7.0 (1.0–18.0) vs. 4 (0.0–12.0), p<0.001].

**Conclusion:**

The presence of T2D and high levels of FPG are independent risk factors for daytime sleepiness and adversely affect sleep quality. Prospective studies addressed to demonstrate whether glycemia optimization could improve the sleep quality in T2D patients seem warranted.

## Introduction

The well-known negative impact of sleep breathing disorders on glucose metabolism has switched to a bidirectional relationship during the last decade, in which type 2 diabetes (T2D) has appeared to be an independent risk factor for both nocturnal intermittent hypoxia and sleep fragmentation [1, 2]. In this regard, the sleeping time spent with oxygen saturation lower than 90% (CT90) is three-fold higher among obese subjects with T2D in comparison with control subjects. In addition, different patterns of sleep disordered breathing have been described in this population, mainly characterized by an increase in apneic events [3, 4]. Furthermore, when metabolic control is improved in patients with T2D it results in a fast and significant reduction in oxygen desaturation events [5]. However, there is a lack of specific studies aimed at investigating whether T2D and metabolic control are also linked to changes in quality of sleep and daytime somnolence.

The problem is of major clinical relevance when we consider that the prevalence of sleep apnea-hypopnea syndrome (SAHS) in subjects with T2D reaches from 40% to 86%, in comparison to the 2 to 4% of symptomatic SAHS in general population [6–8]. Therefore, a growing awareness of the importance of screening and identifying sleep breathing disorders in patients with T2D has emerged in recent years, not only to modify their cardiovascular and metabolic risk profile but also the day to day consequences [9]. However, formal sleep studies in Sleep Units are time consuming and not economically viable for testing entire populations.

Sleep questionnaires have been suggested as a good tool to evaluate not only the risk of having sleep apnea, but also nocturnal abnormalities and the daily consequences related to sleep breathing disorders [10].

However, and although T2D has been sporadically related with daytime sleepiness, it is unknown whether T2D impairs daily somnolence and quality of sleep independently from polysomnographic parameters such as CT90 and apnea-hypopnea index (AHI) [11–14]. In order to shed light to this issue we have designed a case-control study addressed to globally evaluate the deleterious effect of T2D on daily somnolence and sleep quality by means of the administration of specific questionnaires.

## Material and Methods

### Ethics statement

Informed written consent was obtained from all participants, and the human ethics committee of the three participating University Hospitals (Vall d’Hebron, Arnau de Vilanova, and Segovia) approved both the study and the consent procedure.

### Main association

The association of T2D with somnolence symptoms and sleep quality through the administration of specific questionnaires. The degree of glycaemic control was also considered as a covariate with T2D to evaluate the association with related variables.

### Design of the study and description of study population

Somnolence symptoms were evaluated in a first case-control study including 413 patients with T2D and 413 non-diabetic subjects (controls), matched by age, gender, BMI, and waist and neck circumferences. Sleep quality was assesses in a second case-control study including 45 patients with T2D and 135 non-diabetic subjects (controls), matched by age, gender, BMI, and waist circumference (but no neck circumference). The STROBE criteria were followed to report the results [15].

### Description of patients being evaluated for daily somnolence

A total of 1093 patients of Caucasian origin attending the outpatient endocrinology clinic of three university hospitals (Arnau de Vilanova, Vall d’Hebron, and Segovia) were enrolled at the time of a regular visit between January 2010 and December 2014. The degree of sleepiness was evaluated using the Epworth Sleepiness Scale (ESS). Data from an overnight sleep study were available from five hundred and six (62%) of these subjects, more of them obese subjects also evaluated for bariatric procedures.

Inclusion criteria included: age older than 18 years, known T2D for longer than 5 years, blood fasting plasma glucose (FPG) and glycated hemoglobin determinations during the preceding month, the absence of reported nocturnal hypoglycemia or a previous history of severe hypoglycemia, and the ability to read and understand the correct meaning of the questions.

On this basis, among the 498 patients with T2D who met inclusion criteria, 85 were excluded because of the following explanations: missing data (n = 43), chronic pulmonary disease (n = 13) or treatment with continuous positive airway pressure (CPAP) (n = 9), stroke or heart failure (n = 8), alcohol abuse or the use of sedatives (n = 5), active malignancy (n = 4), shift workers (n = 2), and clinical manifestations of diabetic autonomic neuropathy (n = 1). Among non-diabetic subjects, we aimed to select one control for every case and, subsequently, the control group consisted of 413 subjects individually matched to cases by age (within 3-years range), gender, BMI (within 3.5 kg/m^2^ range), waist and neck circumferences, and smoking status. The main clinical characteristics and metabolic data of this population are shown in Table 1.

**Table 1:** Main clinical characteristics and metabolic data of participants included in the study evaluating daytime somnolence. AHI: apnea hypopnea index.

	Type 2 Diabetes	Non Type 2 Diabetes	Mean difference (95% CI)	p value
**N**	413	413	-	-
**Women, n (%)**	215 (52,0)	215 (52,0)	-	-
**Age (years)**	55.9 ± 10.4	55.0 ± 10.0	0.9 (-0,5 to 2.2)	0.712
**BMI (kg/m**^**2**^**)**	36.8 ± 8.1	36.4 ± 9.0	0.4 (-07 to 1.6)	0.458
**Waist circum. (cm)**	115.5 ± 16.0	114.8 ± 17.2	0.7 (-1.9 to 3.3)	0.583
**Neck circum. (cm)**	40.1 ± 5.9	40.8 ± 4.2	-0.7 (-1.7 to 0.3)	0.185
**Glucose (mmol/l)**	9.1 ± 3.1	5.4 ± 0.6	3.7 (3.3 to 4.0)	< 0.001
**HbA1c (%)**	7.9 ± 1.6	5.7 ± 0.4	2.2 (2.0 to 2.4)	< 0.001
**HbA1c (mmol/mol)**	63.4 ± 17.5	38.9 ± 5.2	24.5 (22.4 to 26.5)	<0.001
**AHI (events/hour)** ^a^	25.7 (1.0 to 128.0)	24.1 (0.0 to 118.2)	-	0.060

^a^ Data from an overnight sleep study were only available from 175 patients with type 2 diabetes and 332 controls.

### Description of patients being evaluated for sleep quality

The Pittsburgh Sleep Quality Index (PSQI) questionnaire, which calculates sleep quality and disturbances, was also administered to 331 patients attending the same outpatient clinics between May 2013 and December 2014. Among the 173 patients with T2D who met the same previous inclusion criteria, 38 were excluded because of the following explanations: missing data (n = 27), chronic pulmonary disease or CPAP treatment (n = 5), stroke or heart failure (n = 3), alcohol abuse or the use of sedatives (n = 2), and shift workers (n = 1). We selected one control for every three cases and, subsequently, the control group consisted of 45 subjects without diabetes matched to cases by age (within 7-years range), gender, BMI (within 4.5 kg/m^2^ range), and smoking status. No data from an overnight sleep study were available from these subjects. The main clinical characteristics and metabolic data from this second population are shown in Table 2.

**Table 2:** Main clinical characteristics and metabolic data of the subgroup of participants included in the study evaluating risk of sleep apnea sleep quality.

	Type 2 Diabetes	Non Type 2 Diabetes	Mean difference (95% CI)	p value
**N**	135	45	-	-
**Women, n (%)**	75 (55.5)	25 (55.5)	-	-
**Age (years)**	60.9 ± 12.6	59.0 ± 12.0	1.9 (-2.3 to 6.1)	0383
**BMI (kg/m**^**2**^**)**	30.9 ± 5.3	29.6 ± 7.0	1.4 (-0.6 to 3.3)	0.192
**Waist circum. (cm)**	105.9 ± 14.5	105.0 ± 14.1	0.8 (-4.6 to 6.4)	0.001
**Glucose (mmol/l)**	8.1 ± 2.9	5.3 ± 0.7	2.7 (2.2 to 3.3)	< 0.001
**HbA1c (%)**	7.7 ± 1.7	5.7 ± 0.5	2.0 (1.5 to 2.4)	< 0.001
**HbA1c (mmol/mol)**	61.0 ± 18.7	39.1 ± 6.3	21.9 (17.0 to 26.8)	<0.001

In all cases, after obtaining informed consent, participants were asked about demographic data and medical history. T2D was defined according to the criteria recommended by the Expert Committee on the Diagnosis and Classification of Diabetes [16].

### Specific questionnaires

The ESS was created in 1991, and has become the most common method worldwide to for evaluating the subjective degree of sleepiness. The questionnaire asks respondents to rate their likelihood of falling asleep unintentionally in 8 situations of real-life [17, 18]. The score ranged from 0 (would never doze) to 3 (high chance of dozing), with the higher scores indicate being more sleepy. Daytime sleepiness is considered high when it’s higher than 10.

The PSQI evaluates subjective sleep quality over the preceding month [19]. It is a 19-item questionnaire grouped into seven component scores, each weighted equally on a 0–3 scale, dealing with major aspects of sleep (subjective sleep quality, sleep latency, sleep duration, sleep efficiency, sleep disturbances, use of sleeping medications, and daytime dysfunction). The seven components are then added up to yield a global PSQI score (range: 0–21), where higher scores reflects worse subjective sleep quality, and a PSQI global score greater than 5 identifies poor sleepers.

### Statistical analysis

The normal distribution of the variables was evaluated using the Kolmogorov-Smirnov test. Data were expressed either as the mean ± SD, percentage or median (total range). For parametric tests, AHI, and CT90 were logarithmically transformed to achieve a normal distribution. Comparisons between groups were performed using the Student’s t test or the Mann-Whitney U test for continuous variables, as well as the χ^2^ test for categorical variables.

Regression trees were used to specifically assess the relationship and potential interaction of HbA1c and FPG with ESS, and the minimization of the reduction in the mean squared error was applied as a statistical criterion. Partitions were defined by the systematic exploration of all possible thresholds, and using the Gini criterion to select the on with the highest predictive value. This was performed using a computational intensive algorithm. Therefore, three partitions were defined: (i) HbA1c<6.4% (46.0 mmol/mol); (ii) HbA1c≥6.4% (45.5 mmol/mol) and FPG<13.1 mmol/mol; and (iii) HbA1c≥6.4% (45.5 mmol/mol) and FPG≥13.1 mmol/l. The association of each partition with ESS was then assessed with a Kruskal-Wallis test, and the value of the partition as a predictor of ESS >10 was assessed with a logistic regression model. In addition, the odds-ratios, 95% confidence intervals, and corresponding p-values from a Wald test were calculated. A Receiver Operating Characteristic (ROC) analysis and sensitivity and specificity analyses were also performed.

Furthermore, to evaluate the relationship between age, BMI, waist and neck circumference and polysomnographic measurements with ESS the Pearson linear correlation test was performed. Additionally, stepwise multiple linear regression analyses to explore the variables independently related to the ESS score were also performed. The independent variables included in the analyses were: gender, polysomnographic measurements (AHI and CT90), and the either the presence or absence of T2D or baseline blood glucose levels. All p- values were two-sided, setting the threshold for significance at p<0.05. Statistical analyses were performed using the SSPS statistical package (SPSS, Chicago, IL, USA) and the R statistical package [20].

## Results

The ESS global score was significantly higher in patients with T2D (7.4±4.5 *vs*. 6.5±4.3; p = 0.003), with 23.9% of subjects with T2D presenting an excessive daytime sleepiness (ESS score >10) (23.9% vs. 16.9%, p = 0.016). When all subjects were evaluated, the ESS global score was higher in men than in women (7.5±4.3 vs. 6.5±4.5, p = 0.002), and in those with an HbA1c ≥ 7.0% (53.0 mmol/mol) or FPG ≥ 7.0 mmol/l in comparison with those with lower parameters (7.3±4.7 vs. 6.5±4.1, p = 0.031, and 7.4±4.4 vs. 6.6±4.3, p = 0.018, respectively).

**Fig 1:**
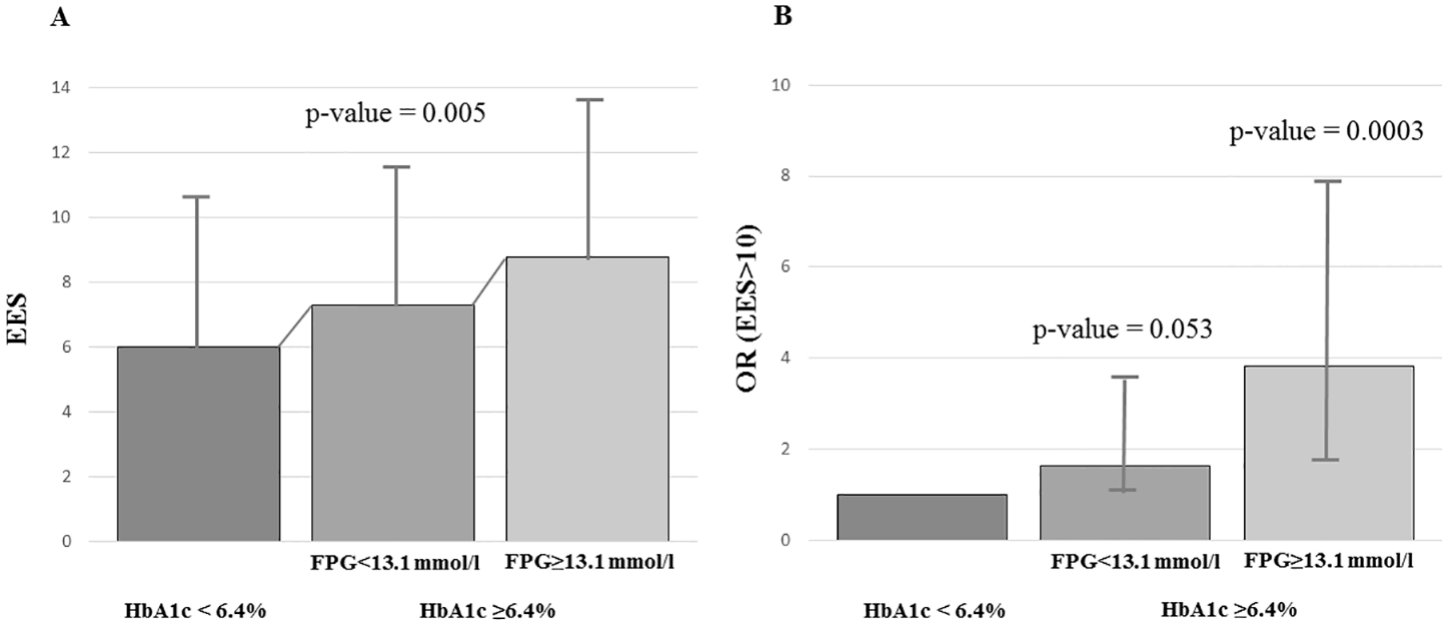
Association of HbA1c and glycemic levels (FPG) with ESS. (A) Patients classified in three different groups according to HbA1c and FPG levels: [HbA1c < 6.4% (46.0 mmol/mol)], [HbA1c ≥6.4% (46.0 mmol/mol) and FPG <13.1 mmol/l] and [HbA1c ≥6.4% (46.0 mmol/mol) and FPG ≥13.1 mmol/l]. Mean ESS levels and standard deviation are shown. (B) Odds-ratios (OR) with corresponding 95% CI to assess the increased risk of excessive daily somnolence (defined as ESS>10) in groups [HbA1c ≥6.4% (46.0 mmol/mol) and FPG <13.1 mmol/l] and [HbA1c ≥6.4% (46.0 mmol/mol) and FPG ≥13.1 mmol/l] with respect to group [HbA1c < 6.4% (46.0 mmol/mol)].

The regression tree analysis revealed a partition of patients into three groups significantly associated to ESS levels (p = 0.005) (Fig 1A). Subjects with HbA1c <6.4% (46.0 mmol/mol) were identified as the lower risk group for EDS (ESS: 6.2±4.2). Meanwhile, among patients with higher degrees of HbA1c, those with FPG<13.1 mmol/l showed an intermediate ESS score (ESS: 7.1±4.4), and those with higher FPG levels were identified as the higher risk group for EDS (ESS 8.6±4.4) (Fig 1A). When specifically assessing the risk of excessive daytime sleepiness (ESS >10) the intermediate group showed an increased risk with respect to the low group close to statistical significance (OR = 1.63 and p = 0.053), and the high risk group showed a clearly significant increase in daytime sleepiness risk (OR = 3.9, 95% CI 1.8–7.9, p = 0.0003) (Fig 1B and Table 3).

**Table 3:** Assessment of the risk of excessive daily somnolence (defined as ESS > 10) in three different groups based on HbA1c and FPG levels. Baseline category (intercept) refers to patients with low HbA1c [<6.4% (46.0 mmol/mol)]. OR (95% CI): odds-ratio with corresponding 95% confidence interval; p-value from a Wald test; beta (SE): estimated parameters and standard deviation from a logistic regression model are shown. ESS: Epworth Sleepiness Scale.

	Beta (SE)	OR (95% CI)	p value
**Intercept [HbA1c < 6.4% (46.0 mmol/mol)]**	-1.76 (0.20)	-	-
**HbA1c ≥6.4% (46.0 mmol/mol) and FPG <13.1 mmol/l**	0.49 (0.25)	1.63 (1.002, 2.72)	0.053
**HbA1c ≥6.4% (46.0 mmol/mol) and FPG ≥13.1 mmol/l**	1.34 (0.37)	3.81 (1.82, 7.91)	0.0003

In the univariate analysis, a correlation between daytime sleepiness and polysomnographic measurements was found (CT90: p = 0.044, r = 0.092; and AHI: p = 0.003, r = 0.130) but we did not find any correlation between ESS score and FPG (p = 0.269, r = 0.043), HbA1c (p = 0.147, r = 0.065), age (p = 0.828, r = 0.008), BMI (p = 0.661, r = -0.015), waist (p = 0.313, r = 0.041) and neck (p = 0.119, r = 0.082) circumferences.

A stepwise regression analysis showed that the presence of T2D (beta = 0.222, p<0.001) and gender (beta = 0.112; p = 0.013), but not polysomnographic parameters (log CT90: beta = 0.057, p = 0.210; log AHI: beta = 0.072, p = 0.118] independently predicted the ESS score (R^2^ = 0.057). In addition, when the presence of T2D was replaced by the FPG, this was the only variable independently associated with ESS score (R2 = 0,012; beta = 0,111, p = 0,028).

On the other hand, the PSQI global score was significantly higher in patients with T2D than in control subjects [7.0 (1.0–18.0) vs. 4 (0.0–12.0), p<0.001]. Almost 2 out of every 3 patients with diabetes were classified as poor sleepers (PSQI global score >5: 67.4% vs. 37.7%, p<0.001), and this subjective sleep quality reduction was associated with higher age (62.5±12.4 vs. 57.0±11.9 years, p = 0.003) and higher FPG (140.6±56.5 vs. 124.5±40.8 mg/dl, p = 0.041), but not with HbA1c or with anthropometric values. In addition, a PSQI global score > 5 was also associated with a higher percentage of patients with an ESS score > 10 (25.4 vs. 12.1%, p = 0.024), with a significative and positive linear correlation between the PSQI and ESS scores (r = 0.214; p = 0.004).

## Discussion

We provide evidence that T2D is associated with impaired scores in different questionnaires that globally evaluate the risk of having daytime somnolence, and sleep quality.

Our results show that excessive daytime sleepiness (EDS) is a common feature in T2D and affects almost one quarter of this population. In a previous work, Bixler et al, in a random sample of 16,583 men and women, identified being treated for depression as the most important risk factor for the complaint of EDS, followed by BMI, age, subjective sleep duration, smoking, sleep apnea, and also T2D [12]. In addition, it has been suggested that subjects with T2D experience dozing when stopped for a few minutes in traffic while driving, increasing their risk for traffic accidents [14].

Increasing attention has to be paid to this data for care providers, as EDS has been previously associated with a general decrease in motivation to participate in activities related to the management of T2D [13]. There are also studies that have failed to demonstrate an association between T2D and EDS. Basta el al. reported in a cohort of 1,106 consecutive patients with sleep apnea that the predictors of EDS were AHI, the lack of regular exercise, and depression, but not the presence of T2D [11]. However, symptoms consistent with sleep apnea were required in order to be included in this series, and therefore this is not comparable with the T2D patients included in our study.

The present study strongly suggests that the presence of T2D is related to EDS independently of other classic risk factors such as SAHS. Our results agree with those previously published by Dixon et al. [21] in 1,055 consecutive obese subjects waiting for bariatric surgery. In this study the ESS global score was higher in men, older patients, and those with T2D, whereas no relationship between daytime somnolence and polysomnography measurements, including the AHI, were reported. In addition, we observed that EDS was evident in those patients with higher FPG levels but an independent relationship with HbA1c was not found. These results suggest a relationship between EDS and immediate or short term glucose fluctuations rather than with sustained glycemic control.

According to the PSQI, more than half of the patients with T2D were “poor sleepers”. Recently, poor sleep quality was proved to increase the risk of the development of T2D in a Chinese adult population during a 5-year follow-up period [22], and the PSQI score has been positively correlated with inflammatory markers such as IL-6 and ICAM-1 [23]. In addition, “poor sleep” has been associated with worse diabetes-related quality of life, a greater number of diabetic complications, and higher levels of depressive symptoms [24]. Similarly, lower sleep quality was associated with poorer glucose control, after controlling for age, gender, BMI, insulin use, and the presence of complications [25]. Several factors could influence the bad sleep quality reported by participants with T2D such as pain due to peripheral neuropathy, nocturia, sleep apnea, and restless leg syndrome, all of them associated with frequent nighttime awakenings contributing to exacerbate sleep disruption [26–28]. In our study sleep quality reduction was associated with basal hyperglycemia but not with HbA1c, strengthening the potential relationship between nocturnal hyperglycemia and sleep quality. Moreover, in our study, sleep quality was associated with daytime sleepiness, thus reinforcing the potential effect of sleep in the daily functioning of patients with T2D.

Some limitations should be taken into account in evaluating the outcome of our study. First, this was an observational study and, therefore, we cannot draw a causal link between T2D or blood glucose levels and either the sleep or the daytime consequences. In this regard, research addressed to determining whether the short term improving of metabolic control ameliorates daytime somnolence and sleep quality is warranted. Conversely, the effect of sleep quality on blood glucose levels should also be examined. Second, as daytime sleepiness often occurs during the postprandial status, a potential relationship between EES and postprandial glucose levels could be speculated. Nevertheless, no data about glucose profiles is available from patients included in this study. Another limiting factor is that sleep characteristics and daytime sleepiness were subjectively assessed, and perhaps a more objective test, i.e. the Multiple Sleep Latency Test (MSL-T), could have found a greater percentage of sleepy patients [29, 30]. In this way, confounding factors like the number of times a subject has to get up for urination or the number of working day hours have not been included in the analysis of results. However, the ESS is used worldwide in routine clinical practice as a standard measure in the evaluation of EDS.

In conclusion, T2D and high levels of FPG are independent risk factors for daytime sleepiness and sleep quality. Prospective studies addressed to demonstrate whether glycemia optimization could improve the sleep quality in T2D patients seems warranted. In addition, our findings emphasize the importance of screening new patients for sleep problems, with a referral to a sleep medicine specialist if appropriate, and suggest sleep hygiene strategies as a part of diabetes management.
